# Safety profile of lutein-based blue dyes and surgical lights

**DOI:** 10.3389/fphar.2025.1704098

**Published:** 2025-11-24

**Authors:** Mario R. Romano, Federica Conti, Francesca Lazzara, Barbara Parolini, Mariantonia Ferrara, Claudio Bucolo

**Affiliations:** 1 Department of Biomedical Sciences, Humanitas University, Milan, Italy; 2 Department of Ophthalmology, Humanitas Gavazzeni-Castelli, Bergamo, Italy; 3 Department of Biomedical and Biotechnological Sciences, School of Medicine, University of Catania, Catania, Italy; 4 Center for Research in Ocular Pharmacology-CERFO, University of Catania, Catania, Italy; 5 Eyecare Clinic, Brescia, Italy; 6 Department of Medical and Surgical Specialties, Radiological Sciences, and Public Health, University of Brescia, Brescia, Italy; 7 School of Medicine, University of Malaga, Málaga, Spain

**Keywords:** LED, xenon, lutein, reactive oxygen species, retinal pigment epithelium

## Abstract

**Introduction:**

The combination of surgical endoillumination and vital dyes has been associated with phototoxicity and retinal damage, despite vital dyes being mainly considered safe. Based on this perspective, the aim of the present study was to evaluate the effect of lutein-based blue dyes (LBBDs) on human retinal pigmented epithelial (ARPE-19) cells and their combination with surgical light exposure.

**Methods:**

ARPE-19 cells were exposed to LBBDs and/or xenon/LED light according to the following experimental design: phase I, exposure to LBBDs only; phase II, exposure to lights only; phase III, single sequential exposure to LBBDs and xenon/LED light; and phase IV, double sequential exposure to LBBDs and xenon/LED light. ATPlite and reactive oxygen species (ROS) detection assays were used to assess the impact on cell viability and ROS production, respectively.

**Results:**

No cytotoxic effect was detected following the exposure to LBBDs for 5 min. LED and xenon lights elicited a cytotoxic effect and an overproduction of ROS for an exposure time of ≥5 min. The ROS overproduction following 5-min exposure of ARPE-19 cells to both LED and xenon lights was significantly (*p* < 0.05) counteracted by pre-treatment with LBBDs. Finally, after double sequential exposure to LBBDs and LED/xenon lights, 2% LBBD induced a significant (*p* < 0.05) reduction in ROS production compared to both LED/xenon exposure and 1% LBBD/light exposure.

**Discussion:**

These findings demonstrated the primary role of light-induced damage as a primary contributing factor to potential retinal damage following peeling procedures. LBBDs provided a protective effect against light-induced oxidative damage, highlighting their potential role in enhancing the safety profile of staining in retinal surgery.

## 1 Introduction

Vital dyes, biocompatible chemical chromophores, are valuable intraoperative tools in vitreoretinal surgery, particularly for aiding the peeling of the internal limiting membrane (ILM) and epiretinal membrane (ERM) (Rodrigues et al., 2009). Traditionally used off-label, green dyes have largely been replaced by blue dyes due to their superior tolerability and safety profile. Among them, trypan blue (TB) and brilliant blue G (BBG) have long been used, either alone or in combination, due to their selectivity for ERM and ILM, respectively (Rodrigues et al., 2009). In recent years, several BBG-derivatives have been specifically developed to enhance ILM selectivity, thereby improving staining efficacy and potentially the biocompatibility of traditional BBG (Spadaro et al., 2020). In this regard, a recent experimental study demonstrated that pure-benzyl BBG (PBB®), a patented BBG-derivative, exhibits high affinity for ILM, mainly through high binding affinity for fibronectin, while showing minimal diffusion into underlying retinal layers (Spadaro et al., 2020). Although blue vital dyes are generally deemed safe, cases of macular toxicity have been reported following macular surgery, and the potential combined role of surgical endoillumination and vital dyes in inducing iatrogenic retinal damage has been advocated (Neffendorf and Jackson, 2025). Intraocular fiberoptic endoilluminators are known to pose a risk of phototoxicity, primarily through photochemical mechanisms (Glickman, 2002). Moreover, as the distance between the light source and the retina is a key factor influencing the risk of damage, macular surgery is considered at higher risk due to the proximity of the light pipe to the posterior pole for precise visualization (Glickman, 2002).

Lutein-based blue dyes (LBBDs) have recently been introduced, with two marketed LBBDs combining 1% soluble lutein +0.05% PBB® and 2% soluble lutein +0.05% PBB® + 0.15% TB, respectively. Lutein is a xanthophyll carotenoid and is one of the major macular pigments in the human retina. It plays a crucial protective role against oxidative stress by filtering high-energy blue light and scavenging reactive oxygen species (ROS) (Gong et al., 2017; Kamoshita et al., 2016). Through its antioxidant properties, lutein stabilizes cellular membranes, prevents lipid peroxidation, and reduces photo-induced oxidative damage in retinal cells (Choo et al., 2022; Gong et al., 2017). This natural pigment may contribute to dye formulations with a retinal neuroprotective effect against light-induced damage, ultimately enhancing their safety profile (Sasaki et al., 2009; Wilson et al., 2021).

In this regard, the growing attention toward the biocompatibility of intraocular medical devices has underscored the crucial role of *in vitro* translational studies in evaluating the safety profile of compounds used intraocularly (Januschowski et al., 2018; Gatto et al., 2021; Romano et al., 2021). Given this context, we performed an experimental *in vitro* study to evaluate the effect of LBBDs and surgical light exposure, both individually and in combination, on human retinal pigmented epithelial (ARPE-19) cells. More specifically, we tested both single and double sequential exposure to each LBBD and two different surgical lights to simulate both the routine single staining and re-staining in more complex surgical scenarios.

## 2 Methods

### 2.1 Cell culture

ARPE-19 cells were purchased from ATCC®. The cell line was cultured at 37 °C (humidified atmosphere with 5% CO_2_) in ATCC-formulated DMEM: F12 medium (ATCC number 30-2006) with 100  U/mL penicillin, 100 μg/mL streptomycin, and 10% fetal bovine serum. The cells were grown to an appropriate density and used at 15–20 passages.

### 2.2 Light sources

The lights tested in this study were as follows:

- LED light (100% blue light and 100% white light), 45 lumens (lm)
- Xenon light (50% and 80%), 80 lumens (lm)

### 2.3 Vital dyes tested

The vital dyes used in the present study were as follows:

− 1% soluble lutein +0.05% PBB® (Single Lutein Blue®/BLutein™ Dye 400, Alfa Instruments, Italy)
− 2% soluble lutein +0.05% PBB® + 0.15% TB (Double Lutein Blue®/BLutein™ Dye 500, Alfa Instruments, Italy)

Hereafter, the above-mentioned dyes will be abbreviated as SLB and DLB, respectively.

### 2.4 Experimental protocol

The study was divided into four phases, which were conducted on different and independent samples:

1. Phase I: Evaluation of the safety profile of SLB and DLB on ARPE-19 cells with the following endpoints: ATPlite and ROS assays after 5-min exposure to 50 µL of each dye formulation without medium, followed by washing with PBS 1X.
2. Phase II: Evaluation of the safety profile of the tested lights. Cells were placed under LED or xenon lamp probes at a distance of 1 mm for different times (100% blue light: 30″, 1′, 5′, 15′, and 30′; 50% xenon light: 1′, 5′, 15′, and 30′) to identify non-toxic and toxic exposure. Controls were left in the dark. After each exposure, ROS intracellular content and ATP release were measured.
3. Phase III a: Evaluation of the safety profile of a single combined exposure to vital dyes and light sources under non-toxic conditions. Specifically, cells were first treated with 50 µL of SLB or DLB for 1′ to mimic a routine staining time. After washing in PBS 1X, cells were irradiated with LED or xenon light probes under non-toxic light conditions (30″ LED and 1′ xenon). After each exposure, ATPlite and ROS levels were assessed.

Phase III b (one shot): Safety profile evaluation of combo exposure to vital dyes and light sources under toxic conditions to better mimic clinical conditions. Specifically, cells were first treated with 50 µL of SLB or DLB for 1′ or 3′ to mimic a routine or prolonged staining time (as can happen in challenging cases, such as highly myopic eyes). After washing in PBS 1X, cells were irradiated with LED or xenon light probes under toxic light conditions (5′ or 15′ LED and 5′ xenon). After each exposure, ATPlite and ROS levels were assessed.

4. Phase IV (double shot): Safety profile evaluation of combo exposure to dyes and light sources to simulate a re-staining clinical scenario. In particular, cells were first treated with 50 µL of SLB or DLB for 1′, and after washing in PBS 1X, they were exposed to LED (100% white light, 5′) or xenon (80%, 5′) light sources. This protocol was carried out twice. After the double exposure, ATPlite and ROS were assessed.

### 2.5 ATPlite assay

We evaluated cell viability by measuring the production of adenosine triphosphate (ATP) using the PerkinElmer ATPlite 1step Luminescence Assay System. After seeding 2.5 × 10^4^ ARPE-19 cells per well in 96-well plates (Costar; Corning, Inc., Corning, NY) and performing the exposure as described above, ARPE-19 cells were washed twice with PBS 1X, and 100 µL of buffer solution (ATPlite) was added to each well according to the manufacturer’s protocol. Following 2-min incubation at room temperature (shaker, 700 rpm), luminescence was evaluated using the Varioskan Microplate Reader (Thermo Fisher Scientific, Waltham, MA). We reported the results as the percentage of the control.

### 2.6 ROS detection assay

The production of ROS was measured to evaluate the oxidative damage induced by the tested compounds/lights. ROS was measured by a 2′,7′-dichlorofluorescein diacetate (DCFDA) Cellular Reactive Oxygen Species Detection Assay Kit (Abcam, Cambridge, United Kingdom). DCFDA, a cell-permeable fluorogenic dye, is deacetylated by cellular esterases to a non-fluorescent compound and later oxidized by ROS to highly fluorescent 2′,7′-dichlorofluorescein (DCF); fluorescence intensity is proportional to the ROS concentration of the cells. After seeding and treatment as above, ARPE-19 cells were stained by adding 100 µL/well of the diluted DCFDA solution (25 µM). Cells were incubated with this solution for 45 min at 37 °C in the dark. After removing the DCFDA solution, 100 µL/well of 1X buffer was added, and ROS concentration was measured immediately by detecting DCF fluorescence (λ_ex_ = 495 nm and λ_em_ = 529 nm) using a Varioskan™ Flash Multimode Reader. The results were reported as the percentage of the control after background subtraction; to determine total ROS formation, fluorescence was normalized to the fluorescent intensity of control, untreated cells.

### 2.7 Statistical analysis

Statistical analysis was performed using Prism 10 (GraphPad, San Diego, CA). The data generated by all the experiments were reported as the mean ± SD (n = 6). One-way analysis of variance (ANOVA) was carried out, and Tukey’s *post hoc* test was used for multiple comparisons. Differences between groups were considered statistically significant at *p* < 0.05.

## 3 Results

### 3.1 Phase I: Lutein-based blue dyes are well-tolerated by ARPE-19 cells

**FIGURE 1:**
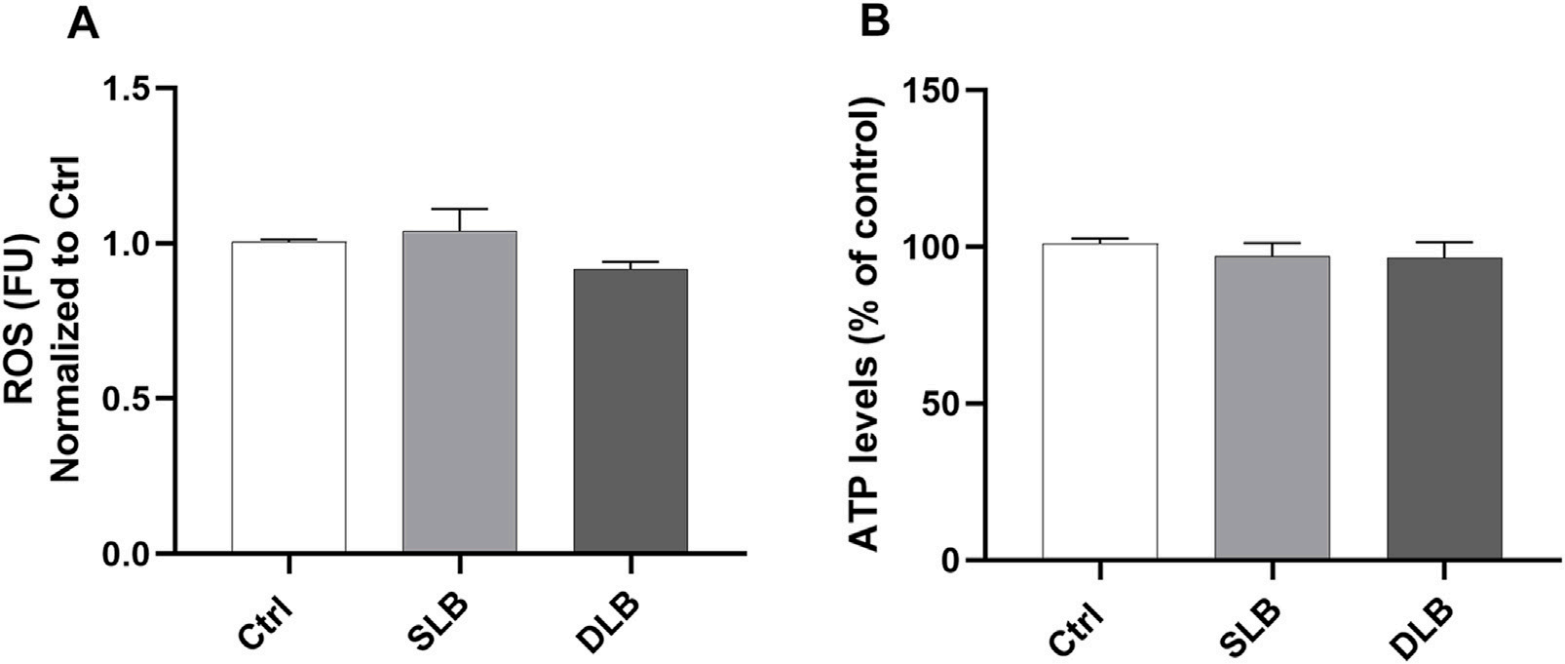
ARPE-19 cells’ tolerability to SLB (1% soluble lutein + 0.05% PBB®) and DLB (2% soluble lutein + 0.05% PBB® + 0.15% trypan blue). ROS **(A)** and ATPlite **(B)** assays were carried out after 5 min of treatment with vital dyes. Each bar represents the mean value ± SD (n = 6; each run in triplicate). Data were analyzed by one-way ANOVA, and Tukey *post hoc* test was performed for multiple comparisons.

After 5 min of exposure, neither SLB nor DLB significantly influenced cell viability or ROS production (Figure 1).

### 3.2 Phase II: Prolonged exposure to LED and XENON lights induces cytotoxicity in ARPE-19 cells

**FIGURE 2:**
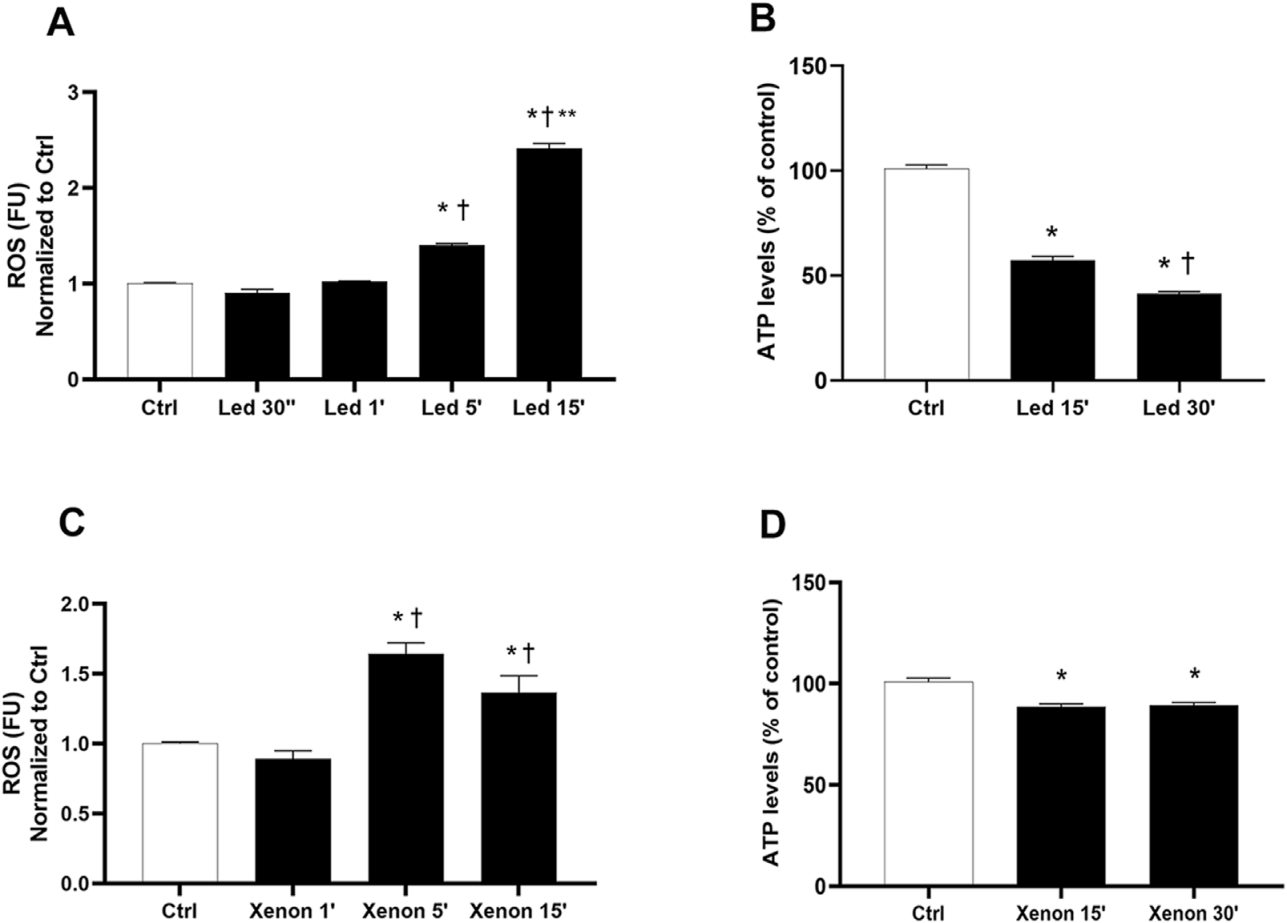
Time-dependent effect of LED and xenon light sources on ARPE-19 cells. **(A)** ROS levels were evaluated after 30 s and 1, 5, and 15 min of led exposure. **(B)** ATPlite was carried out after 15 and 30 min of exposure to LED probes. **(C)** ROS levels were evaluated after 1, 5, and 15 min of xenon exposure. **(D)** ATPlite was carried out after 15 and 30 min of exposure to xenon probes. Each bar represents the mean value ± SD (n = 6; each run in triplicate). Data were analyzed by one-way ANOVA, and Tukey *post hoc* test was performed for multiple comparisons. *p < 0.05 vs. ctrl; †*p* < 0.05 vs. LED 15’ (panel B), LEd 30″ and 1’ (panel A), xenon 1’ (panel C); **p < 0.05 vs. LED 5’.

Figure 2 shows the effect of LED light exposure. In particular, exposure to LED lights induced a significant reduction in ATP levels (*p* < 0.05) in a time-dependent manner (Figure 2B). Regarding ROS production, 30″ and 1 min of LED exposure did not induce a significant increase in ROS levels (Figure 2A); conversely, a significant (*p* < 0.05) time-dependent overproduction of ROS was observed after 5 and 15 min, not only compared to control cells but also compared to the other exposure times (30″ and 1’; Figure 2A). Based on these data, 30″ of LED exposure was selected as the non-toxic condition and 5′ and 15′ were selected as toxic conditions; 30 min of exposure was excluded for the subsequent experiments. After exposure of ARPE-19 cells to xenon light for 15 and 30 min, a similar significant (*p* < 0.05) reduction in ATP levels was detected (Figure 2D). As observed in Figure 2C, 1-min exposure to xenon light did not induce ROS production; on the other hand, after 5 and 15 min, ROS production was significantly (*p* < 0.05) increased compared to that in both control cells and 1′-irradiated cells (Figure 2C). Regarding xenon light, 1 and 5 min represent the non-toxic and toxic conditions, respectively.

### 3.3 Phase III a: Pre-treatment with LBBDs under non-toxic light conditions increased ROS production

**FIGURE 3:**
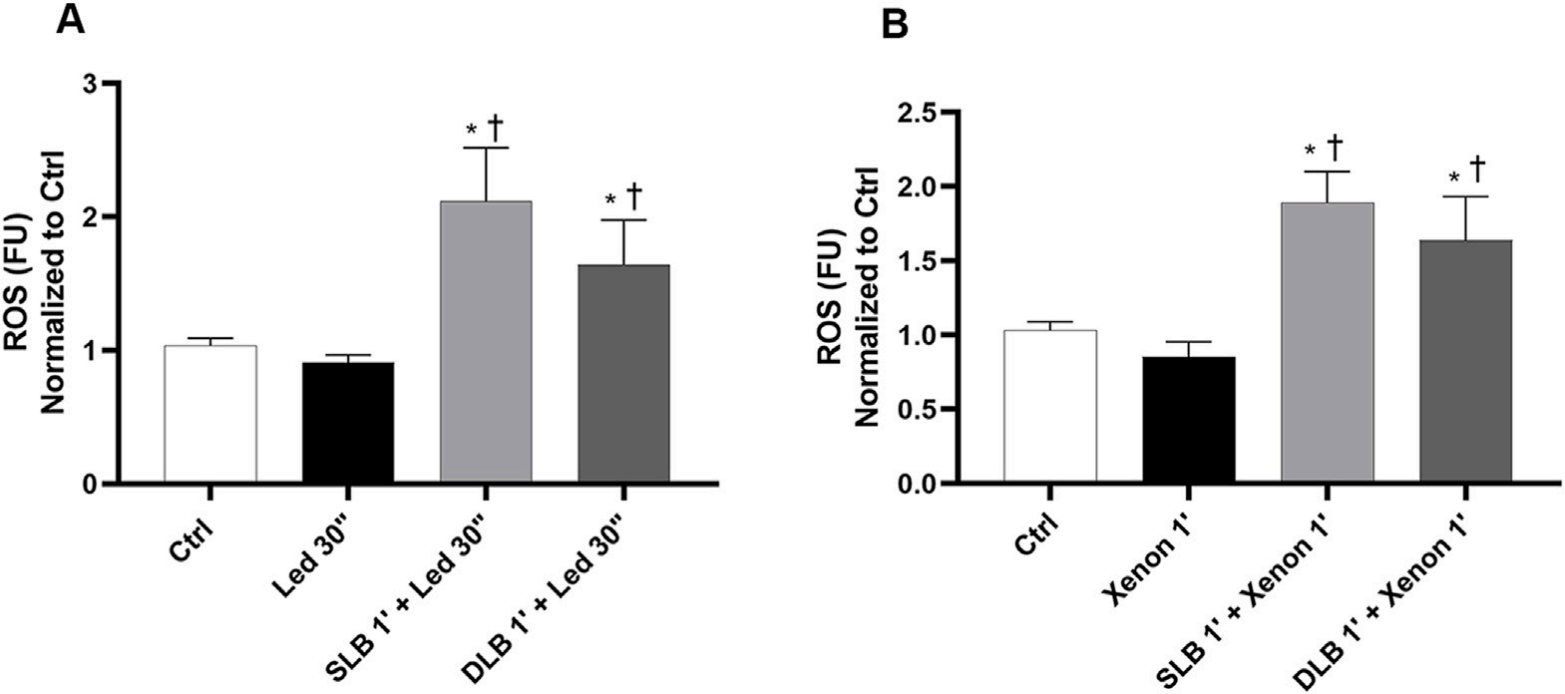
ROS assay after exposure to vital dyes, LED and xenon lights under non-toxic conditions. ROS assay was carried out after 1 min of treatment with vital dyes, followed by 30 s and 1 min of exposure to LED **(A)** and xenon **(B)** probes. SLB (single lutein blue, 1% soluble lutein +0.05% PBB®); DLB (double lutein blue, 2% soluble lutein +0.05% PBB® + 0.15% trypan blue). Each bar represents the mean value ±SD (n = 6; each run in triplicate). Data were analyzed by one-way ANOVA, and Tukey *post hoc* test was performed for multiple comparisons. *p < 0.05 vs. ctrl; †*p* < 0.05 vs. LED 30’’ (panel A) and xenon 1’ (panel B).

LED and xenon light exposure for 30″ and 1′, respectively, did not increase intracellular ROS production compared to that in non-exposed control cells, as shown in Figure 3. Under these non-toxic conditions, ROS production increased significantly (*p* < 0.05) when ARPE-19 cells were pre-treated for 1 min with SLB or DLB and then irradiated with LED light for 30’’ (Figure 3A) or xenon light for 1’ (Figure 3B) compared to that when both cells were exposed to LED and xenon light only and the controls.

### 3.4 Phase III b: Pre-treatment with LBBDs counteracts the increased ROS production induced by lights in toxic conditions

**FIGURE 4:**
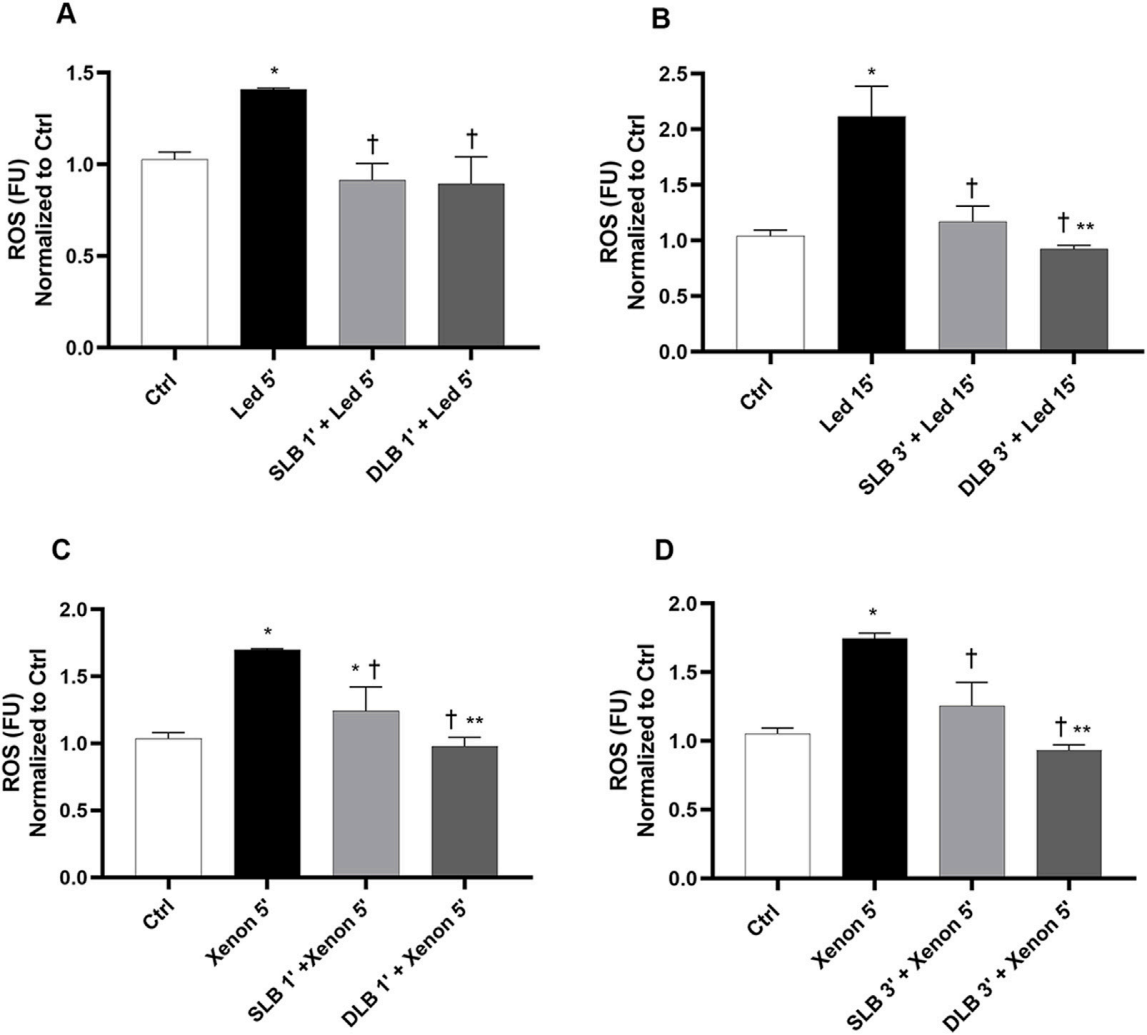
ROS assay after single-exposure to vital dyes, LED, xenon light under toxic conditions. ROS assay was carried out after 1 min of treatment with vital dyes, followed by exposure for 5 min to LED **(A)** or xenon **(C)** lights. In panel **(B)** (led 15′) and **(D)** (xenon 5′), ROS assay was carried out after 3 min of treatment with vital dyes, followed by exposure to light. SLB (1% soluble lutein +0.05% PBB®); DLB (2% soluble lutein +0.05% PBB® + 0.15% trypan blue). Each bar represents the mean value ±SD (n = 6; each run in triplicate). Data were analyzed by one-way ANOVA, and Tukey *post hoc* test was performed for multiple comparisons. **p < 0.05 vs*.* Single lutein blue 3’+ LED 15’ (panel B), single lutein blue 1’+ Xenon 5’ (panel C), and single lutein blue 3’+ xenon 5’ (panel D).

The intracellular ROS increase induced by toxic exposure to LED light for 5 min was significantly (*p* < 0.05) counteracted by both LBBDs after 1 min of pre-treatment (Figure 4A). In addition, as shown in Figure 4B, irradiation with LED light under toxic conditions (15′) induced a significant (*p* < 0.05) increase in ROS levels compared to that in non-exposed control cells. The pre-treatment for 3 min with vital dyes significantly (*p* < 0.05) protected ARPE-19 cells from LED-light-induced damage (Figure 4B). Moreover, ARPE-19 cells pre-treated with DLB for 3 min and then exposed to LED light (15′) showed a significant (*p* < 0.05) decrease in ROS production compared to those pre-treated with SLB (Figure 4B).

Five minutes of xenon exposure (toxic condition) significantly (*p* < 0.05) increased ROS production compared with that in the control group (Figures 4C,D). However, when cells were pre-treated with vital dyes (1′) and then exposed to xenon light (5′), ROS production was significantly (*p* < 0.05) reduced compared to that in ARPE-19 cells exposed only to xenon light (Figure 4C). Additionally, ROS levels after DLB treatment were significantly lower than ROS levels of cells treated with SLB (Figure 4C). The pre-treatment for 3 min with both vital dyes significantly (*p* < 0.05) protected ARPE-19 cells from 5′ xenon-light-induced damage (toxic condition) (Figure 4D). As for LED lights, 3′ of pre-treatment with DLB significantly reduced (*p* < 0.05) ROS levels, not only compared with those in xenon-exposed cells but also compared with those in cells exposed to SLB/xenon (Figure 4D). The ATPlite assay was carried out in both phase III a and III b, and no differences among the groups of treatment were detected (Supplementary Figures S1,S2).

### 3.5 Phase IV: LBBDs exert a protective effect after double exposure to LBBDs and lights

**FIGURE 5:**
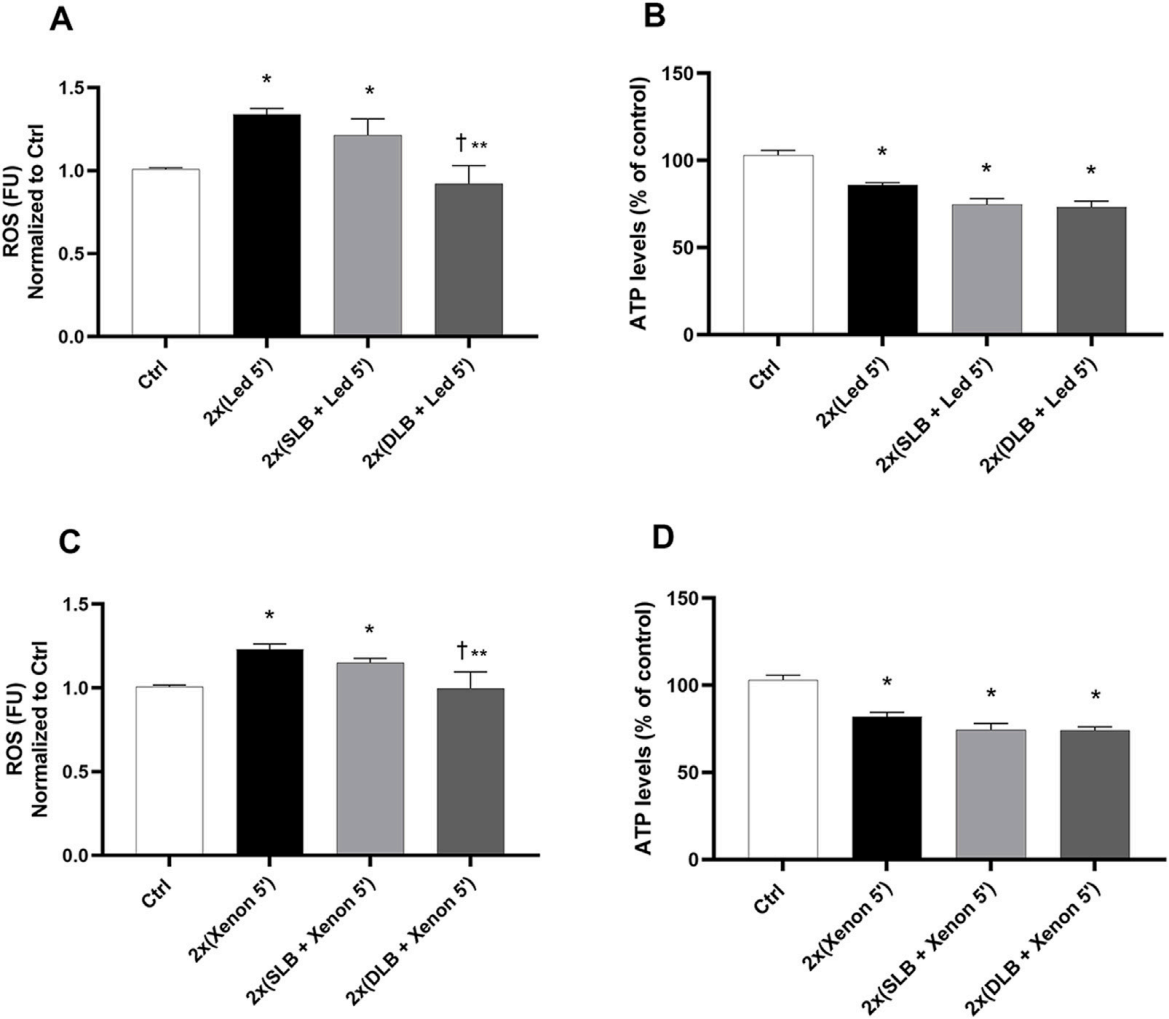
ROS and ATPlite assays after double treatment with vital dyes and double exposure to LED or xenon light under toxic conditions. **(A)** ROS and ATPlite **(B)** assays were carried out after 1 min of treatment with vital dyes, followed by 5 min exposure to LED probes, 1 min of treatment with vital dyes, and then 5 min of exposure to LED light. **(C)** ROS and ATPlite **(D)** assays were carried out after 1 min of treatment with vital dyes, followed by 5 min exposure to xenon probes, 1 min of treatment with vital dyes, and then 5 min of exposure to xenon light. SLB (1% soluble lutein +0.05% PBB®); DLB (2% soluble lutein +0.05% PBB® + 0.15% trypan blue). Each bar represents the mean value ±SD (n = 6; each run in triplicate). Data were analyzed by one-way ANOVA, and Tukey *post hoc* test was performed for multiple comparisons. *p < 0.05 vs. ctrl; ^†^ *p*<0.05 vs. 2 x (LED 5′) (panel A), 2 x (xenon 5′) (panel C); **p<0.05 vs. 2 x (single lutein blue + LED 5′) (panel A), 2 x (single lutein blue + xenon 5′) (panel C).

As shown in Figure 5, double exposure to LED or xenon probes significantly increased (*p* < 0.05) ROS production compared to that in control cells (not exposed) (Figures 5A,C). Double 1-min exposure to DLB and 5-min LED/xenon exposure significantly reduced (*p* < 0.05) ROS production compared to that in cells exposed only to LED/xenon light (5’+5′) and to cells treated with SLB (Figure 5). Regarding ATP, double exposure to both LED and xenon light for 5′ led to a significant reduction in ATP levels compared to that in control non-exposed cells (Figures 5B,D). The combination of double treatment with SLB and DLB (1’+1′) and double lights exposure (5’+5′) did not significantly impact ATP reduction compared to that with LED/xenon alone.

## 4 Discussion

**FIGURE 6:**
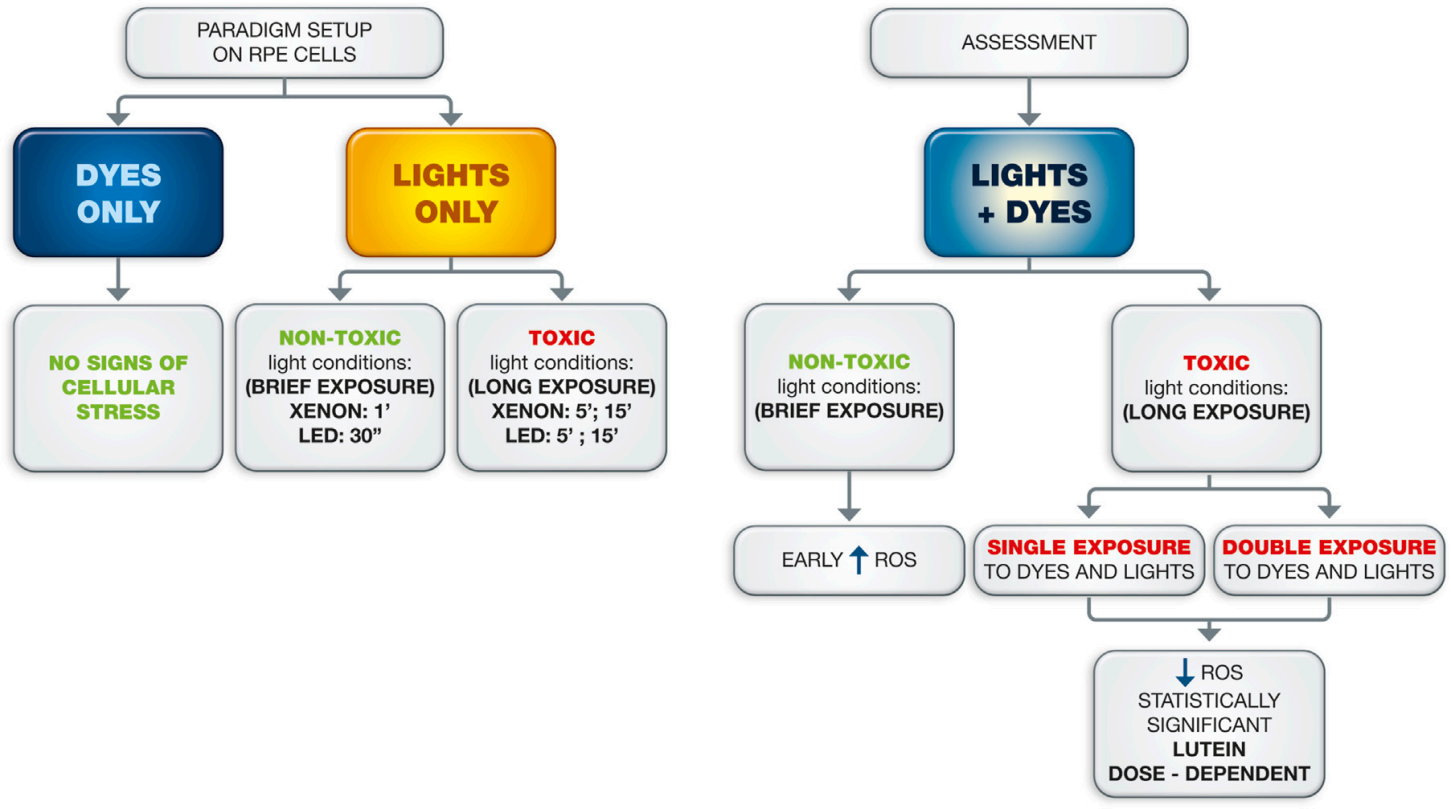
Representation of the different experimental steps and results. The diagram illustrates the main steps of the study: the paradigm setup on ARPE-19 cells, evaluating the effect of dyes and lights alone, and the assessment of vital dyes and lights combination. The arrows indicate the timeline of the procedures and the relationships between the different workflow steps.

The results of the present study, summarized in Figure 6, showed the protective role of lutein-based blue dyes against the damage elicited by light during retinal surgery. Several cases of severe macular toxicity following both ILM and ERM peeling using BBG, TB, or combined blue dyes have been reported (Al-Halafi, 2013). Concerns have recently emerged regarding the potential retinal toxicity of blue dyes and surgical lights related to the exacerbation of phototoxic damage and oxidative stress (Costa et al., 2013).

Different mechanisms have been hypothesized to explain dye-induced toxicity, such as i) partial overlap between the spectra of dye absorbance and light emission (Costa et al., 2009), ii) formation of ROS and free radicals due to a light-induced dye decomposition (Al-Halafi, 2013), and iii) photosensitizing effect of the dye (Narayanan et al., 2005). Notably, due to their non-fluorescent nature, blue dyes are expected to contribute remarkably less to phototoxicity than green dyes (Awad et al., 2018).

In this context, it is crucial to identify the main factor responsible for retinal damage by exploring the effects of light alone, dye alone, and their combination. *In vitro* cytotoxicity tests play an important role not only in assessing the safety profile of single compounds but also in evaluating the potential interaction between different intraoperative agents (Gatto et al., 2021; Lazzara et al., 2023). On this basis, we investigated the effects of two LBBDs, surgical lights (LED and xenon), and their combination using different exposure times to better mimic various surgical scenarios.

Both tested LBBDs were well-tolerated by ARPE-19 cells, as evidenced by the absence of significant changes in cell viability and ROS production. This finding aligns with previous experimental studies on lutein combined with other vital dyes (Casaroli-Marano et al., 2015). The tested LBBDs, composed of soluble lutein + PBB® ± TB, were specifically developed to enhance both the staining efficacy and safety profile of the final dye formulation (Romano et al., 2018; Spadaro et al., 2020). The combination of PBB®s increased ILM selectivity, and the protective and antioxidant properties of lutein are expected to improve retinal defense, particularly in procedures where the retina is at high risk of phototoxic damage, such as macular surgery.

Regarding surgical lights, both LED and xenon lights induced a significant decrease in cell viability and an increase in ROS production after exposure time ≥5 min. The formation of free radicals and oxidative stress are key contributors to photochemical damage, exerting detrimental effects on both the neurosensory retina and the RPE, (Verma et al., 2001; Glickman, 2002; Youssef et al., 2011). Among the light-related factors influencing the risk of phototoxicity (e.g., intensity, wavelength, and distance from the tissue), the duration of light exposure is well-established (Glickman, 2002; Chalam et al., 2015), which is consistent with the time-dependent effect observed in our experiments. From a clinical perspective, these findings explain the higher risk of retinal toxicity associated with prolonged peeling maneuvers as this condition involves prolonged exposure to endoillumination at a short distance. These results reinforce the primary role of phototoxicity in chromo-vitrectomy-induced damage (Neffendorf and Jackson, 2025).

In the third experimental phase, we performed the sequential exposure of cells to dyes and lights, mimicking a real surgical scenario. Under non-toxic light conditions (e.g., 30″ LED/1 min xenon), ARPE-19 cells probably rely primarily on their endogenous antioxidant defenses in order to maintain low ROS levels. When ARPE-19 cells are pre-treated with lutein-based vital dye and then exposed to light, lutein, being a scavenger, may remain inactive as it requires the presence of reactive species to exert its antioxidant function. Blue dyes (trypan blue and brilliant blue G derivative), acting as photosensitizers, could absorb light energy and undergo photoexcitation. This process might lead to the early production of ROS via type-I and type-II photochemical mechanisms (Glickman, 2002). Instead, under toxic conditions (prolonged light exposure), oxidative stress could act as a trigger for lutein activation, quenching the ROS. This condition could explain the observed paradoxical effect. The protective effect of lutein is evident after long light exposure, when its interaction with ROS is sustained and functionally relevant. During extensive oxidative stress, accumulation of lutein in the RPE cells preserves the proteasome from inactivation. Preservation of proteasome inactivation by lutein is one of the mechanisms through which this pigment may modulate the inflammatory response to photo-oxidative stress (Bian et al., 2012; Prathyusha et al., 2025). Accordingly, when toxic LED/xenon conditions induced significant oxidative stress (remarkable ROS overproduction), the pre-exposure to both LBBDs mitigated this detrimental effect. Furthermore, with longer dye exposure (3 min), the positive effect on ROS production was significantly greater when testing the dye with a higher lutein concentration (2%, DLB). These findings support a beneficial protective effect of LBBDs against light-induced oxidative damage, which is in line with the antioxidant properties of lutein, which can inhibit the lipoperoxidation of membranes (Sasaki et al., 2009; Wilson et al., 2021). This beneficial effect also appears to persist in the case of re-staining. In phase 4 of the experiment, the sequential exposure to dye and lights was repeated to simulate the re-staining procedure in more challenging cases. In this context, not only did DLB induce a significant decrease in ROS production but pre-exposure to both LBBDs also had no significant impact on cell viability compared to the group exposed to light only. This further suggests that light exposure is the primary contributor to chromo-vitrectomy-related retinal damage.

Overall, these results reinforce previous clinical studies reporting the advantages of LBBDs in ophthalmic surgical procedures (Badaro et al., 2014; Maia et al., 2014; Romano et al., 2018). In particular, a faster functional recovery and lower immunohistochemical expression of Muller cell markers have been reported in eyes where LBBDs were used to aid ERM/ILM peeling compared to those when using indocyanine green and a combined TB/BBG dye (Romano et al., 2018). It was speculated that these findings, suggesting a reduced peeling-associated damage, may be attributed to the reduced adhesion of LBBDs to the retinal surface due to a different interaction with membranes (Sousa-Martins et al., 2015; Romano et al., 2018). This *in vitro* model provides a valid and reproducible approach for investigating phototoxic and photochemical effects under surgical-like conditions in retinal pigment epithelial cells. Nevertheless, despite this model mimicking surgical conditions (i.e., the time of exposure to vital dyes and to lights), it lacks the physiological complexity of the *in vivo* retinal environment.

In conclusion, our findings support the primary role of light-induced damage as the primary contributing factor to potential retinal damage following peeling procedures. Furthermore, the results suggest that staining with LBBDs does not cause any damage under toxic conditions of light exposure. On the contrary, LBBDs appear to provide a protective effect against light-induced oxidative damage, especially DLB, through their higher percentage of lutein than SLB, highlighting their potential role in enhancing the safety profile of staining in retinal surgery.

## Data Availability

The raw data supporting the conclusions of this article will be made available by the authors, without undue reservation.

## References

[B1] Al-Halafi A. M. (2013). Chromovitrectomy: update. Saudi J. Ophthalmol. 27, 271–276. 10.1016/j.sjopt.2013.10.004 24371423 PMC3841301

[B2] Awad D. Wilińska J. Gousia D. Shi X. Eddous J. Müller A. (2018). Toxicity and phototoxicity in human ARPE-19 retinal pigment epithelium cells of dyes commonly used in retinal surgery. Eur. J. Ophthalmol. 28, 433–440. 10.1177/1120672118766446 29607665

[B3] Badaro E. Furlani B. Prazeres J. Maia M. Lima A. A. S. Souza-Martins D. (2014). Soluble lutein in combination with brilliant blue as a new dye for chromovitrectomy. Graefes Arch. Clin. Exp. Ophthalmol. 252, 1071–1078. 10.1007/s00417-013-2539-5 24441951

[B4] Bian Q. Gao S. Zhou J. Qin J. Taylor A. Johnson E. J. (2012). Lutein and zeaxanthin supplementation reduces photooxidative damage and modulates the expression of inflammation-related genes in retinal pigment epithelial cells. Free Radic. Biol. Med. 53, 1298–1307. 10.1016/j.freeradbiomed.2012.06.024 22732187 PMC3744865

[B5] Casaroli-Marano R. P. Sousa-Martins D. Martínez-Conesa E. M. Badaró E. Nunes R. P. Lima-Filho A. A. (2015). Dye solutions based on lutein and zeaxanthin: *in vitro* and *in vivo* analysis of ocular toxicity profiles. Curr. Eye Res. 40, 707–718. 10.3109/02713683.2014.952831 25153042

[B6] Chalam K. V. Li W. Koushan K. Grover S. Balaiya S. (2015). Effect of distance and duration of illumination on retinal ganglion cells exposed to varying concentrations of brilliant blue green. J. Clin. Med. Res. 7, 517–524. 10.14740/jocmr2085e 26015816 PMC4432893

[B7] Choo P. P. Woi P. J. Bastion M.-L. C. Omar R. Mustapha M. Md Din N. (2022). Review of evidence for the usage of antioxidants for eye aging. BioMed Res. Int. 2022, 5810373. 10.1155/2022/5810373 36225983 PMC9550496

[B8] Costa E. de P. F. Rodrigues E. B. Farah M. E. Dib E. Penha F. (2009). Vital dyes and light sources for chromovitrectomy: comparative assessment of osmolarity, pH, and spectrophotometry. Invest Ophthalmol. Vis. Sci. 50, 385–391. 10.1167/iovs.08-2285 18689696

[B9] Costa E. F. Barros N. M. T. Coppini L. P. Neves R. L. Carmona A. K. Penha F. M. (2013). Effects of light exposure, pH, osmolarity, and solvent on the retinal pigment epithelial toxicity of vital dyes. Am. J. Ophthalmol. 155, 705–712. 10.1016/j.ajo.2012.10.004 23253911

[B10] Gatto C. Romano M. R. Giurgola L. Ferrara M. Ragazzi E. D’Amato Tothova J. (2021). *Ex vivo* evaluation of retinal cytotoxicity after the use of multiple medical devices in pars plana vitrectomy in porcine eyes. Exp. Eye Res. 213, 108837. 10.1016/j.exer.2021.108837 34774490

[B11] Glickman R. D. (2002). Phototoxicity to the retina: mechanisms of damage. Int. J. Toxicol. 21, 473–490. 10.1080/10915810290169909 12537644

[B12] Gong X. Draper C. S. Allison G. S. Marisiddaiah R. Rubin L. P. (2017). Effects of the macular carotenoid lutein in human retinal pigment epithelial cells. Antioxidants Basel, Switz. 6 (4), 100. 10.3390/antiox6040100 29207534 PMC5745510

[B13] Januschowski K. Irigoyen C. Pastor J. C. Srivastava G. K. Romano M. R. Heimann H. (2018). Retinal toxicity of medical devices used during vitreoretinal surgery: a critical overview. Ophthalmologica 240, 236–243. 10.1159/000488504 30001544

[B14] Kamoshita M. Toda E. Osada H. Narimatsu T. Kobayashi S. Tsubota K. (2016). Lutein acts *via* multiple antioxidant pathways in the photo-stressed retina. Sci. Rep. 6, 30226. 10.1038/srep30226 27444056 PMC4957151

[B15] Lazzara F. Conti F. Ferrara M. Lippera M. Coppola M. Rossi S. (2023). Safety profile of Lutein- *versus* triamcinolone acetonide-based vitreous staining. Transl. Vis. Sci. Technol. 12, 5. 10.1167/tvst.12.1.5 36598459 PMC9832719

[B16] Maia M. Furlani B. A. Souza-Lima A. A. Martins D. S. Navarro R. M. Belfort R. (2014). Lutein: a new dye for chromovitrectomy. Retina 34, 262–272. 10.1097/IAE.0b013e3182a0b7f4 23917540

[B17] Narayanan R. Kenney M. C. Kamjoo S. Trinh T.-H. T. Seigel G. M. Resende G. P. (2005). Toxicity of indocyanine green (ICG) in combination with light on retinal pigment epithelial cells and neurosensory retinal cells. Curr. Eye Res. 30, 471–478. 10.1080/02713680590959312 16020280

[B18] Neffendorf J. E. Jackson T. L. (2025). Presumed phototoxicity from macular vital staining with brilliant blue G and Trypan Blue: a post-market surveillance study, systematic review, and synthesis of the literature. Surv. Ophthalmol. 70, 339–351. 10.1016/j.survophthal.2024.11.006 39566564

[B19] Prathyusha P. Viswanathan G. Tomcy A. T. Binitha P. P. Bava S. V. Sindhu E. R. (2025). Lutein and inflammation: a comprehensive review of its mechanisms of action. Explor. Drug Sci., 100885. 10.37349/eds.2025.100885

[B20] Rodrigues E. B. Costa E. F. Penha F. M. Melo G. B. Bottós J. Dib E. (2009). The use of vital dyes in ocular surgery. Surv. Ophthalmol. 54, 576–617. 10.1016/j.survophthal.2009.04.011 19682624

[B21] Romano M. R. Ilardi G. Ferrara M. Cennamo G. Parolini B. Mariotti C. (2018). Macular peeling-induced retinal damage: clinical and histopathological evaluation after using different dyes. Graefes Arch. Clin. Exp. Ophthalmol. 256, 1573–1580. 10.1007/s00417-018-4029-2 29948176

[B22] Romano M. R. Ferrara M. Nepita I. D’Amato Tothova J. Giacometti Schieroni A. Reami D. (2021). Biocompatibility of intraocular liquid tamponade agents: an update. Eye (Lond) 35, 2699–2713. 10.1038/s41433-021-01596-w 34035489 PMC8452761

[B23] Sasaki M. Ozawa Y. Kurihara T. Noda K. Imamura Y. Kobayashi S. (2009). Neuroprotective effect of an antioxidant, lutein, during retinal inflammation. Invest Ophthalmol. Vis. Sci. 50, 1433–1439. 10.1167/iovs.08-2493 18997089

[B24] Sousa-Martins D. Caseli L. Figueiredo M. C. Sa E Cunha C. Mota-Filipe H. Souza-Lima A. (2015). Comparing the mode of action of intraocular lutein-based dyes with synthetic dyes. Invest Ophthalmol. Vis. Sci. 56, 1993–2000. 10.1167/iovs.14-16187 25698703

[B25] Spadaro A. Rao M. Lorenti M. Romano M. R. Augello A. Eandi C. M. (2020). New brilliant blue G derivative as pharmacological tool in retinal surgery. Front. Pharmacol. 11, 708. 10.3389/fphar.2020.00708 32523529 PMC7261835

[B26] Verma L. Venkatesh P. Tewari H. K. (2001). Phototoxic retinopathy. Ophthalmol. Clin. North Am. 14, 601–609. 10.1016/s0896-1549(05)70260-1 11787740

[B27] Wilson L. M. Tharmarajah S. Jia Y. Semba R. D. Schaumberg D. A. Robinson K. A. (2021). The effect of Lutein/Zeaxanthin intake on human macular pigment optical density: a systematic review and meta-analysis. Adv. Nutr. 12, 2244–2254. 10.1093/advances/nmab071 34157098 PMC8634499

[B28] Youssef P. N. Sheibani N. Albert D. M. (2011). Retinal light toxicity. Eye (Lond) 25, 1–14. 10.1038/eye.2010.149 21178995 PMC3144654

