# Supplementary material for: Safety profile of lutein-based blue dyes and surgical lights
Source: Front Pharmacol. 2025 Nov 24;16:1704098. doi: 10.3389/fphar.2025.1704098 (PMC12683236; doi:10.3389/fphar.2025.1704098)
Supplement: Supplementary file 1 [file Supplementaryfile1.docx]

Supplementary Material


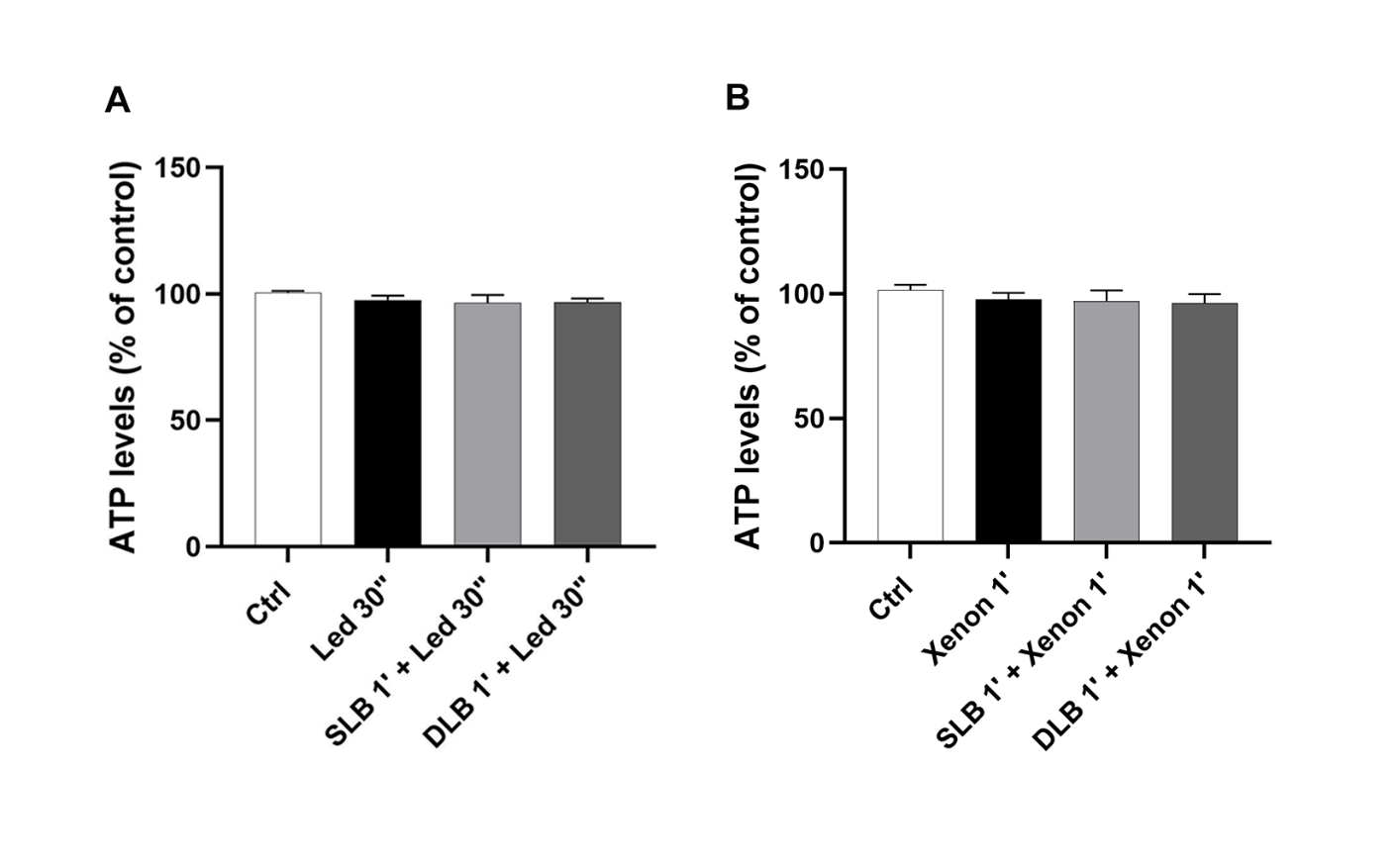
**Supplementary Figure 1. ATPlite assay after exposure to vital dyes, led and xenon lights in non-toxic conditions.** ATPlite assay was carried out after 1 minute of vital dyes treatment, followed by 30 seconds and 1 minute to led **(A)** and xenon **(B)** probes exposure. SLB (single lutein blue, 1% soluble lutein + 0.05% PBB®); DLB (double lutein blue 2% soluble lutein + 0.05% PBB® + 0.15% trypan blue). Each bar represents the mean value ± SD (n=6; each run in triplicate).


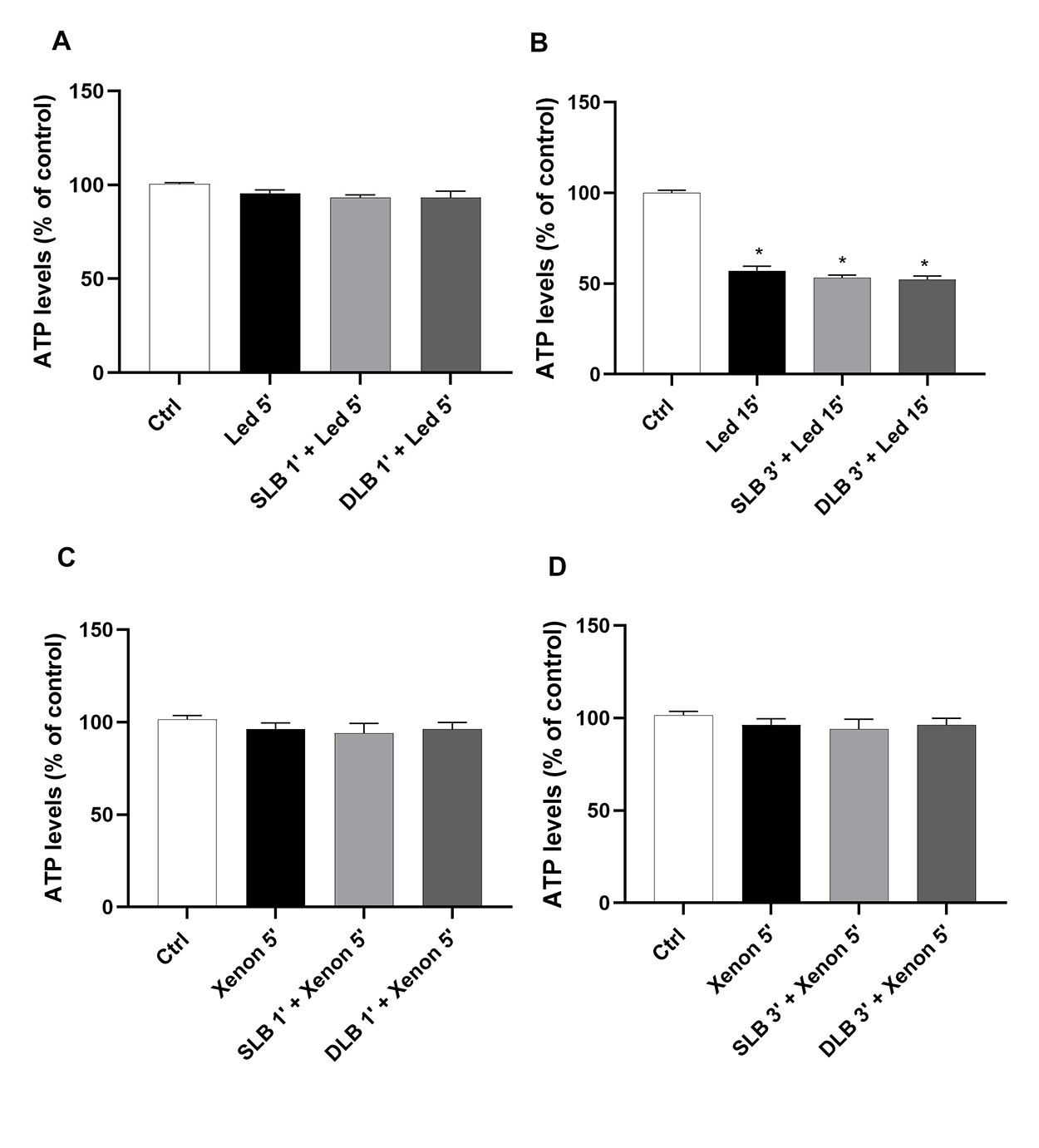


**Supplementary Figure 2. ATPlite assay after single-exposure to vital dyes, led and xenon light in toxic conditions.** ATPlite assay was carried out after 1 minute of vital dyes treatment, followed by exposure for 5’ to led **(A)** or xenon **(C)** lights. In panel **B** (led 15’) and **D** (xenon 5’), ATPlite was carried out after 3 minutes of treatment with vital dyes, followed by lights exposure (15’ led and 5’ xenon). SLB (single lutein blue, 1% soluble lutein + 0.05% PBB®); DLB (double lutein blue, 2% soluble lutein + 0.05% PBB® + 0.15% trypan blue). Each bar represents the mean value ± SD (n=6; each run in triplicate). Data were analysed by one-way ANOVA, and Tukey post-hoc test for multiple comparisons. *p<0.05 *vs* ctrl;
